# Total Intravenous Anesthesia Using Remimazolam for Transcarotid Approach Transcatheter Aortic Valve Replacement: A Case Report

**DOI:** 10.7759/cureus.64628

**Published:** 2024-07-16

**Authors:** Atsuhiro Kitaura, Hiroatsu Sakamoto, Shota Tsukimoto, Haruyuki Yuasa, Yasufumi Nakajima

**Affiliations:** 1 Anesthesiology, Kindai University, Osaka, JPN; 2 Dental Anesthesiology, Kanagawa Dental University, Yokosuka, JPN

**Keywords:** anesthesia, cerebral infarction, remimazolam, transcatheter aortic valve replacement, transcarotid

## Abstract

The transcarotid approach was introduced in Japan in April 2024 as an alternative approach for transcatheter aortic valve replacement (TAVR). Because carotid artery blood flow is reduced on one side during the procedure, there is a risk of intraoperative brain stroke. Therefore, it is crucial to check for cerebral complications immediately after the procedure. We report a case involving an 87-year-old female who underwent transcarotid TAVR under general anesthesia with remimazolam and remifentanil. The operation was completed in a short period. There was no evidence of hypotension during the induction of anesthesia, and intraoperative blood pressure control was easy. However, there was a decrease in local oxygen saturation for approximately nine minutes intraoperatively. Following the administration of flumazenil, the patient was quickly awakened, and neurological findings were confirmed to be normal. The patient was discharged without complications. Our findings suggest that remimazolam, an ultra-short-acting benzodiazepine, is feasible for the transcarotid TAVR procedure due to its minimal circulatory impact and ability to facilitate rapid and reliable arousal.

## Introduction

The transcarotid approach is gaining recognition as the most promising alternative approach for transcatheter aortic valve replacement (TAVR) [1]. The reason for this is that the transcarotid approach is considered to have a relatively low risk of cerebral embolization and is comparable to transfemoral [1]. However, the incidence of cerebral infarction in TAVR is approximately 2-5% [1-3], and cerebral infarction is still a complication that should never be ignored. Because carotid artery blood flow is reduced on one side during the procedure, there is a period of interruption of carotid blood flow [1]. As there is a risk of intraoperative brain stroke, it is important to check for cerebral complications immediately after the procedure. Patients with severe aortic stenosis are more prone to hemodynamic instability during induction, delayed postoperative awakening due to advanced age, and cognitive decline [2]. Remimazolam, an ultra-short-acting benzodiazepine, is considered feasible for the transcarotid TAVR (TC-TAVR) procedure due to its several advantages minimal circulatory impact, and ability to facilitate rapid and reliable arousal [4]. We have been interested in the features of remimazolam since its launch and have used it in many TAVR cases. Our experience has shown that TAVR anesthesia with remimazolam is easy to maintain hemodynamics, allows for rapid and reliable arousal with the use of antagonists, and is relatively easy to maintain spontaneous respiration even at general anesthetic doses [5]. Therefore, we decided to use general anesthesia with remimazolam, which we are familiar with, for this first case of TC-TAVR. To the best of our knowledge, this is the first report of TC-TAVR anesthetized with remimazolam. Herein, we report our present case with a review of the literature.

## Case presentation

**Figure 1:**
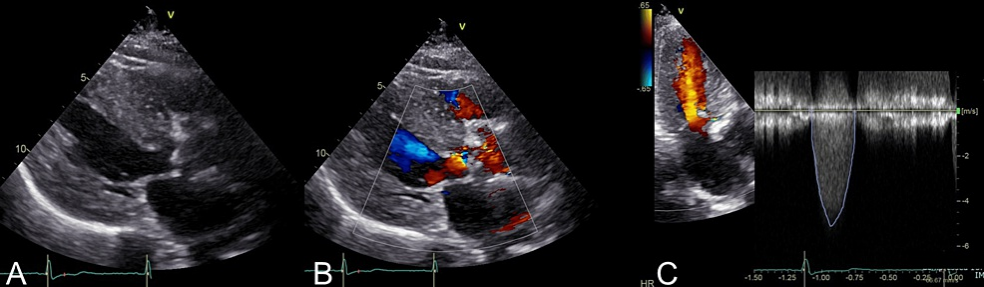
Preoperative transthoracic echocardiography of the present case A) Left ventricular long-axis view (B mode); B) left ventricular long-axis view (Color Doppler mode); C) continuous wave Doppler of aortic valve blood flow (apical 3-chamber view) B-mode showed an aortic valve with calcification and restricted opening. Color Doppler mode showed accelerated blood flow due to aortic valve stenosis. Continuous wave Doppler of aortic valve blood flow revealed the maximum velocity was 5.1 m/sec.

An 87-year-old woman (height: 153 cm, weight: 58 kg) with aortic valve stenosis was referred to our hospital for TAVR. The patient's usual blood pressure was about 150 mmHg systolic. She had a history of hypertension and was taking 5 mg amlodipine and 10 mg propranolol. Her clinical frailty scale was 6. Her echocardiography showed severe aortic stenosis (aortic valve area: 0.84 cm^2^, Vpeak: 5.1 m/s) (Figure 1) and preserved left ventricular ejection fraction of 0.67.

**Figure 2:**
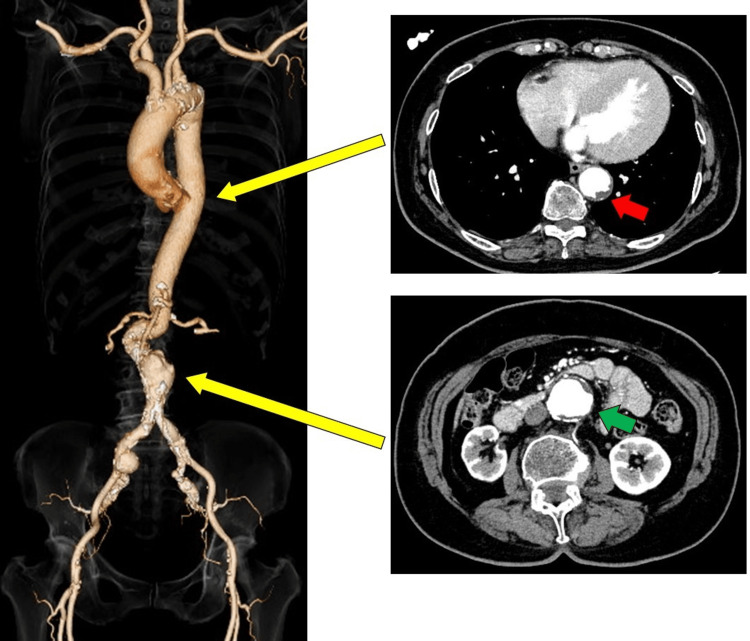
Computed tomography of the aorta of the present case The figure on the left is a 3D reconstructed image of the patient's aorta. The carotid artery had minimal calcification and enough diameter. The abdominal aorta with severe tortuosity and aneurysms were observed. The two figures on the right are axial cross-sectional images, showing representative findings that determined that transfemoral access was unsuitable. Yellow arrows indicated where each axial section corresponds to in the 3D reconstructed image. The red arrow indicated plaques in the descending aorta. The green arrow indicated abdominal aortic aneurysm and atherosclerotic plaque.

Computerized tomography angiography revealed that the vessels were unsuitable for a femoral approach. Specifically, findings included a tortuous abdominal aorta with an aneurysm and a shaggy aorta, bilateral common femoral arteries with calcific stenosis, and a vasculature diameter of less than 5 mm. Her carotid artery diameter was over 6 mm with no calcification, making her suitable for a carotid approach (Figure 2).

**Figure 3:**
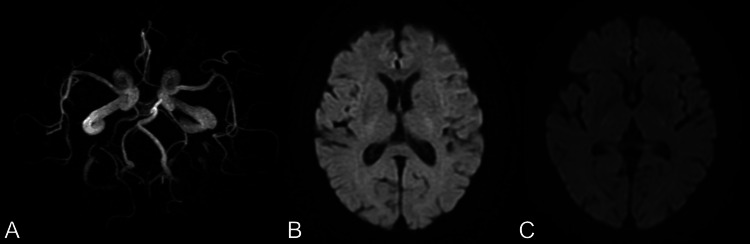
The brain magnetic resonance imaging (MRI) of the present case A) Preoperative magnetic resonance angiography (MRA); B) preoperative diffusion-weighted image (DWI); C) postoperative diffusion-weighted image (DWI) Preoperative MRA showed no defects in the major vessels and the circle of Willis. Preoperative DWI showed no significant lesions except for old lacunar infarction. Postoperative DWI showed no obvious new lesions.

Magnetic resonance angiography showed normal major cerebral arteries and a circle of Willis (Figure 3).

Our heart team decided to perform TAVR via the right carotid approach.

**Figure 4:**
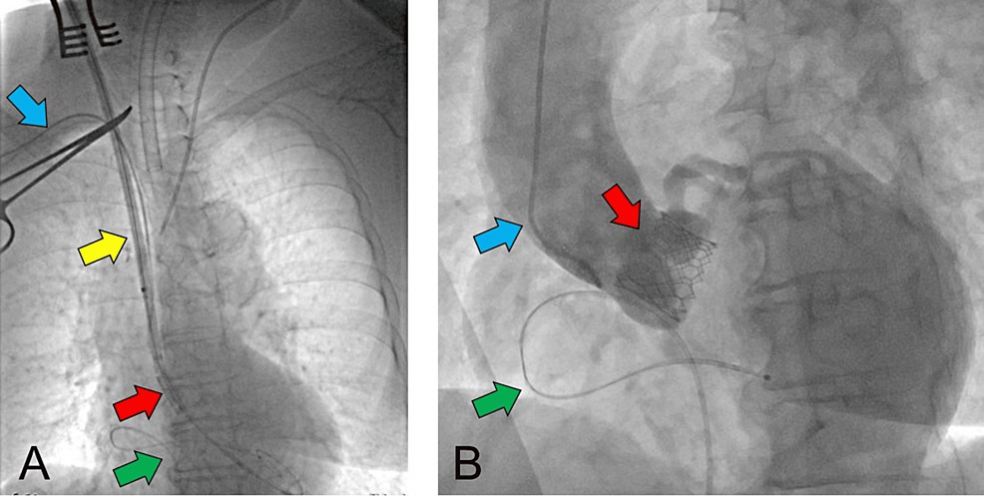
Intraoperative fluoroscopic images A) Fluoroscopic image just before valve-cross; B) post-implantation aortography In Figure 4A, an E-sheath and a pre-shaped stiff wire inserted under direct view through the right carotid artery and an indwelling device that passed through the E-sheath were shown. A contrast catheter was observed near the aortic root from the right radial artery, a pacemaker wire inserted from the right femoral vein in the right ventricle, and A central venous catheter placed from the left internal jugular vein was observed in Figure 4A. Figure 4B shows aortography after device removal. The ideally positioned TAVR valve could be seen. The AR was trivial, but there were no other abnormal findings. Red arrows indicated the TAVR valve (Sapien 3 Ultra RESILIA #23). The yellow arrow indicated the E sheeth. Green arrows showed a pacing catheter placed in the right ventricle. Blue arrows indicated the contrast catheter inserted through a right radial artery.

We opted for general anesthesia. After inserting an arterial pressure line in the patient's left radial artery under local anesthesia, the patient was rapidly induced with 0.1 mg/kg of remimazolam and 0.05 mg of remifentanil and intubated with 0.6 mg/kg of rocuronium. Intraoperatively, the patient was maintained on remimazolam 1 mg/kg/h and remifentanil 0.05 µg/kg/min. The depth of anesthesia was maintained to keep the bispectral index values within the range of 40 to 60. Blood pressure was maintained with noradrenaline at 0.1 to 0.3 µg/kg/min, targeting a systolic blood pressure of 140 mmHg or higher from before the E-sheath insertion until the completion of carotid angioplasty. Regional cerebral oxygen saturation (rSO_2_) was monitored in the bilateral forehead area. As blood pressure rose, rSO_2_ increased; however, the surgical technique temporarily made maintaining the target blood pressure difficult, causing rSO_2_ on the left side to fluctuate with the changing blood pressure. After inserting the E-sheath into the right carotid artery, rSO_2_ (measured with INVOS 5100C, Medtronic, USA) on the right frontal head decreased. Following the device cross, rSO_2_ on the right side had decreased by more than 20% from baseline. However, the Sapien 3 Ultra RESILIA #23 (Edwards Lifesciences Corp., USA) was placed smoothly (Figure 4).

**Figure 5:**
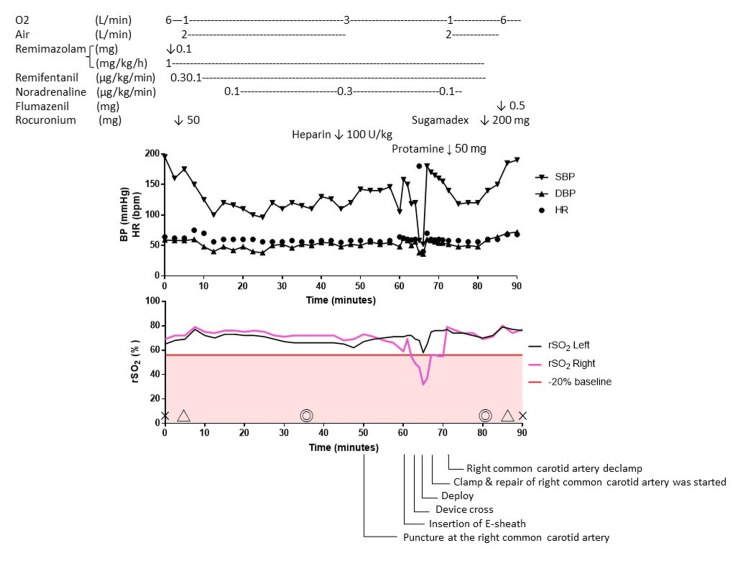
The anesthesia record and the changes in intraoperative rSO2 The top section displayed drug administration, the middle section showed changes in blood pressure and heart rate, and the bottom section illustrated the fluctuations in rSO_2_ measured on the left and right frontal regions. Event timings during the procedure were indicated outside the graphs. Vital signs remained generally stable throughout the procedure. However, there was a downward trend in rSO_2_ on the operative side after the sheath insertion, accompanied by temporary decreases of more than 20% from baseline, coinciding with decreases in blood pressure associated with surgical maneuvers. Cross marks: the beginning and end of anesthesia; Triangles: intubation and extubation; Double circles: the beginning and end of surgery; BP: blood pressure; SBP: systolic arterial blood pressure; DBP: Diastolic arterial blood pressure; HR: heart rate; rSO_2_: regional oxygen saturation

The carotid artery was clamped before the removal of the E-sheath. The carotid artery was unclamped after carotid artery repair. After declamping of the right carotid artery, rSO_2 _quickly recovered. It took approximately 10 minutes from the insertion of the E-sheath to release the carotid artery clamp. rSO_2_ decreased to less than 20% of baseline for approximately nine minutes. Remimazolam and remifentanil were discontinued simultaneously with carotid de-clamping. Fourteen minutes after the end of anesthetic administration, Sugammadex 4 mg/kg and flumazenil 0.5 mg were administered. One minute after administration of the antagonists, the patient awoke and was extubated. Immediately after extubation, the patient was able to speak and perform antigravity movements of the extremities. The anesthesia time was 90 minutes, the operation duration was 46 minutes. Figure 5 illustrates the anesthesia record and the fluctuations in intraoperative rSO_2_.

The patient was admitted to the ICU. The patient left the ICU on postoperative day 1 and was discharged on postoperative day 4. No delirium was noted during hospitalization using confusion assessment methods for the ICU. Cognitive function, assessed using the Revised Hasegawa Dementia Scale (HDS-R), was unchanged from preoperatively (preoperative score versus postoperative score: 26 versus 26). A magnetic resonance imaging performed on postoperative day 2 showed no obvious cerebral infarction (Figure 3C).

## Discussion

In the present case, a small amount of noradrenaline was used to maintain the targeted hemodynamics under adequate general anesthesia. It has been reported remimazolam has a significantly lower incidence of hypotensive events when compared to propofol, the most commonly used intravenous anesthetic [4]. This feature of remimazolam was considered advantageous in the anesthesia of patients with severe aortic stenosis. In fact, a study comparing it to dexmedetomidine-based deep sedation in transfemoral TAVR (TF-TAVR) also reported that it was able to provide nearly equivalent circulatory dynamics [5]. Remimazolam use is also relatively easy to maintain spontaneous respiration, even when used for deep sedation in TAVR patients [5]. Therefore, remimazolam seems likely to be used for sedation of TC-TAVR.

On the other hand, intraoperative patient discomfort was expected to be higher in TC-TAVR than in TF-TAVR because surgical procedures are performed in the neck. It was also considered less stressful for the surgeon to be provided with an immobile surgical field because of the inclusion of surgical procedures such as angioplasty. For these reasons, general anesthesia with remimazolam was chosen for this case because of its minimal effect on cardiac circulation.

TAVR is increasingly indicated for the treatment of patients with severe aortic stenosis, and the number of cases is increasing [6]. Despite the great technological advances, cerebrovascular events remain one of the most feared complications, increasing the risk of morbidity and mortality in the short and long term [7]. The carotid approach is used in TAVR patients for whom the femoral artery approach is unsuitable. Fewer cerebral infarctions have been reported with the carotid approach than with the subclavian approach [1]. The carotid approach is considered to have the second-best prognosis after the femoral artery approach [8]. The incidence of ipsilateral stroke in carotid approach surgeries in TAVR was reported to be low [3]. Therefore, it is currently the second-line approach method in TAVR, following the femoral artery approach. However, even in TF-TAVR, the incidence of cerebral infarction exists in 2.3-4.2% of cases [1-3], most of them acute and related to the procedure [3]. Therefore, intraoperative cerebral infarction is a complication that should not be ignored. In addition, the carotid approach results in decreased or interrupted blood flow in the common carotid artery during the procedure; thus, there is a risk of ipsilateral cerebral ischemia in addition to the risk of vertebral arterial embolism. Indeed, in studies utilizing magnetic resonance imaging, a significant number of TC-TAVR patients were found to have at least one cerebral ischemic lesion on the ipsilateral hemisphere (84.6%) and the contralateral hemisphere (63.5%). The number of ischemic lesions per patient was observed to be higher in the ipsilateral hemisphere compared to the contralateral hemisphere [9]. Although the majority of cerebral ischemic lesions from TAVR are considered asymptomatic, they have been associated with cognitive dysfunction [10]. To preserve cerebral perfusion, it is recommended that body blood pressure be maintained above 140 mmHg systolic [11], but no reliable method of dealing with this problem has been established so far. Currently, it is considered acceptable to substitute a similar technique in carotid endarterectomy [12]. Cerebral blood flow is monitored by measuring rSO_2_ using a near-infrared spectroscopy (NIRS) monitor, as correlations between NIRS measurements and middle cerebral artery blood flow have been reported [13]. Optimal cutoff values for rSO_2_ are a 20% reduction from baseline [14]. In a study of macrovascular surgery using NIRS monitoring, it was reported that a drop in rSO_2_ of more than five minutes is likely to produce neurologic events [15]. In the present case, rSO_2_ was more than 20% below baseline for about nine minutes, as the surgical technique made it difficult to completely maintain the target blood pressure. Carotid artery angioplasty may take longer in some cases, in which case the carotid clamping time will be prolonged. Thus, cerebral infarction may occur in TC-TAVR. It is necessary to wake the patient early after the procedure so that neurological findings can be observed to confirm the presence or absence of cranial nerve complications. On the other hand, elderly patients who undergo TAVR are prone to delayed arousal [16]. Inhaled anesthetics have been found to remain in the body for several hours [17]. Moreover, sensitivity to propofol is increased by 30-50% [16]. Remimazolam is an ultra-short-acting benzodiazepine sedative that provides rapid and reliable arousal due to the presence of flumazenil, an antagonist with an almost equivalent half-life [5,18]. Even without administering an antagonist, remimazolam can achieve awakening in a similar timeframe to propofol due to its short-acting duration. However, we chose to administer flumazenil considering the following potential benefits. Flumazenil has been reported to antagonize the anesthetic effects of remimazolam within approximately one minute [5]. Therefore, it enables reliable and rapid awakening even in elderly patients [5]. This reliable awakening could potentially aid in confirming neurological symptoms, negating serious postoperative neurological complications soon after surgery, and potentially reducing operating room stay and postoperative monitoring efforts.

Interestingly, while benzodiazepine use in elderly patients is typically linked to postoperative delirium in intensive care settings [19], this was not observed in the current case and did not lead to an increase in postoperative delirium [20]. However, additional studies are warranted to investigate the mechanism by which remimazolam is less likely to cause delirium. We believe that there might be one less reason to avoid the use of remimazolam, even in elderly patients.

## Conclusions

We share our experience in a TC-TAVR case managed with total intravenous anesthesia using remimazolam. Our findings suggest that remimazolam, an ultra-short-acting benzodiazepine, is feasible for the TC-TAVR procedure due to its minimal circulatory impact and ability to facilitate rapid and reliable arousal.
